# Selective Deionization of Thin-Layer Samples Using Tandem Carbon Nanotubes–Polymeric Membranes

**DOI:** 10.1021/acs.analchem.3c02965

**Published:** 2023-10-10

**Authors:** Alexander Wiorek, Maria Cuartero, Gastón A. Crespo

**Affiliations:** † Department of Chemistry, School of Engineering Science in Chemistry, Biochemistry and Health, KTH Royal Institute of Technology, Teknikringen 30, SE-114 28 Stockholm, Sweden; ‡ UCAM-SENS, Universidad Católica San Antonio de Murcia, UCAM HiTech, Avda. Andres Hernandez Ros 1, 30107 Murcia, Spain

## Abstract

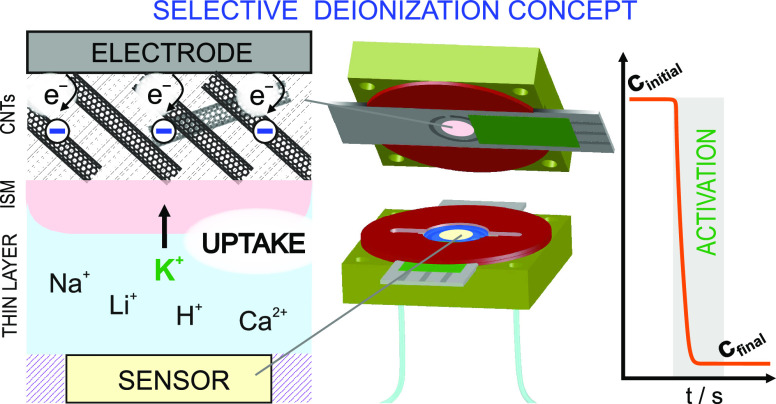

Herein, we investigate the selective deionization (i.e., the removal of ions) in thin-layer samples (<100 μm in thickness) using carbon nanotubes (CNTs) covered with an ionophore-based ion-selective membrane (ISM), resulting in a CNT-ISM tandem actuator. The concept of selective deionization is based on a recent discovery by our group (Anal. Chem.2022, 94, (21), , 7455−74593557954710.1021/acs.analchem.2c00797PMC9161223), where the activation of the CNT-ISM architecture is conceived on a mild potential step that charges the CNTs to ultimately generate the depletion of ions in a thin-layer sample. The role of the ISM is to selectively facilitate the transport of only one ion species to the CNT lattice. To estimate the deionization efficiency of such a process, a potentiometric sensor is placed less than 100 μm away from the CNT-ISM tandem, inside a microfluidic cell. This configuration helped to reveal that the selective uptake of ions increases with the capacitance of the CNTs and that the ISM requires a certain ion-exchanger capacity, but this does not further affect its efficiency. The versatility of the concept is demonstrated by comparing the selective uptake of five different ions (H^+^, Li^+^, Na^+^, K^+^, and Ca^2+^), suggesting the possibility to remove any cation from a sample by simply changing the ionophore in the ISM. Furthermore, ISMs based on two ionophores proved to achieve the simultaneous and selective deionization of two ion species using the same actuator. Importantly, the relative uptake between the two ions was found to be governed by the ion–ionophore binding constants, with the most strongly bound ion being favored over other ions. The CNT-ISM actuator concept is expected to contribute to the analytical sensing field in the sense that ionic interferents influencing the analytical signal can selectively be removed from samples to lower traditional limits of detection.

Nanostructured materials such as carbon nanotubes (CNTs) are today widely used in different applications related to energy storage, desalination, and electrochemical sensors. In particular, differently synthesized CNTs are among the most used ion-to-electron transducers in all-solid-state potentiometric sensors based on polymeric ion-selective membranes (ISM).^1^ Owing to their relatively large capacitance, CNTs were found to be ideal candidates to stabilize the readout of the potentiometric sensors,^2^ minimizing the need for frequent recalibration.^1^ This capacitive behavior has been demonstrated by the results from various techniques, such as electrochemical impedance spectroscopy (EIS),^2^ chronopotentiometry,^3^ and synchrotron radiation-X-ray photoelectron spectroscopy.^4^

Certain types of functionalization allow for tuning/controlling some of the CNTs’ properties. For example, by incorporating redox couples covalently attached to the CNTs’ surface,^5^ the stability of the potential at the transducer–membrane interface of potentiometric sensors was significantly improved, enhancing the long-term stability. This outcome is indeed promising for the transition that potentiometric sensors are today experiencing toward automatized and decentralized measurements. Indeed, much effort has been put into bringing analytical portable devices from the lab to the field for evaluation of environmental water,^6^ point-of-care applications, and sports technology,^7^ among others.

In amperometric and voltammetric sensors, CNTs were used for the detection of hydrogen peroxide^8^ and dopamine,^9^ among other analytes. The improved electrochemical performance observed with CNT-based electrodes was first hypothesized to be an electrocatalytical effect, but later, it was found to originate from (i) impurities in the CNTs’ structure (remaining from their synthesis)^10−12^ or (ii) the intrinsic porosity of the film.^13^ In the latter case, the porosity seems to create a series of thin-layer domains where the analyte exhaustively reacts. This process was found to be very quick, occurring when a sufficient overpotential is reached and resulting in a very characteristic voltametric response: absence of diffusion tail once the redox peak is developed, and a minimal peak separation between the anodic and cathodic waves (i.e., the typical thin layer voltammogram instead of those expected at semi-infinite conditions). This effect was recently rationalized by the Compton group.^14^

Regarding the use of CNTs for deionization purposes, the main focus has been directed to water treatment for the production of drinking water.^14^ The principle of tuning the charging of the double layer of CNT-based electrodes allowed for a massive number of ions to be removed from water.^15^ Notably, some claims about turning this principle selective for several ions by implementing a surface modification of the CNTs have been made.^16^ For example, by the attachment of negatively or positively charged groups to the CNTs, the performance of deionization was improved from the electrostatic forces at the CNT–water interface attracting the oppositely charged ion.^16^ While this approach cannot discriminate between positive and negative ions, it seems good enough for some applications (such as the provision of drinking water and wastewater treatment).

Aiming for a genuine selective deionization, we have recently presented in a letter the combination of CNTs with an ISM for the selective uptake of K^+^ over Na^+^ in aqueous samples.^17^ Now, we rationalize this concept in terms of the roles of the CNT and ISM elements in the ion uptake capabilities. Accordingly, we demonstrate how to maximize the relative uptake and provide the concept with versatility for any cation (e.g., K^+^, Na^+^, Li^+^, H^+^, and Ca^2+^). Additionally, by adding two ionophores into the same ISM, simultaneous uptake of two ion species is possible. To demonstrate all this, the CNT-ISM tandem is integrated in a microfluidic device together with a potentiometric sensor (selective for each of the cations) that monitors the ion uptake in situ and in real-time. Importantly, the sensor was placed face-planar to the actuator, less than 100 μm away from it. Overall, the results suggest a fully capacitive process for the CNTs’ uptake of the ion for which the ISM is selective. Many sensing concepts (including optical and electrochemical readouts) may benefit from this approach because the selective and close-to-exhaustive removal of ions is now possible. Effectively, the CNT-ISM tandem has the potential to be integrated in new sensor–actuator systems to cover certain unaddressed analytical challenges, as discussed in this paper.

## Experimental Section

### The Actuator–Sensor System

To prepare the actuator, first, a mask (Mylar-sheet, RS-components) was placed on the screen-printed electrode (DRP150, Dropsens) using a double-adhesive tape (3M 9471LE, 0.058 mm thick). The mask and the tape were both cut using a Silhouette Cameo cutter (USA). The mask allowed the working electrode (part of the Dropsens) to be independently modified, without affecting the reference and counter electrodes. A dispersion of CNTs in THF (0.5 mg/mL) was prepared using ultrasonification for 1 h, whereafter several additions of 5 μL of the suspension were added onto the electrode surface (by drop casting) to achieve the desired CNT loading, reported through the paper as mg of CNTs per cm^2^. Between each addition of CNTs to the electrode surface, the THF was allowed to evaporate for ca. 1 min. Unless specified, a loading of 0.80 mg CNTs/cm^2^ (corresponding to 40 additions of 5 μL) was used. Then, the screen-printed electrodes modified with CNTs were rotated (1500 rpm, 60 s) while a volume of 20 μL of the corresponding ISM cocktail (see Table S1) was spin coated. Finally, the mask was carefully removed, and the actuator was inserted into the microfluidic cell.

For the potentiometric sensors, three layers of Mylar sheets (two of them were 75-μm thick and another was 100-μm thick), with adhesive tape in between them and making a total mask with a thickness of 425 μm, were used. The mask was attached to the screen-printed electrode to modify the working electrode without affecting the reference electrode. Then, the CNT suspension (10 × 2 μL) was drop casted on the working electrode part and, after drying for 1 h, the membrane was drop casted (5 × 10 μL of the corresponding cocktail). The membrane was left to dry for at least 6 h, being later conditioned overnight in the respective solution (10 mM concentration for each cation and 10 mM acetate buffer for pH). Finally, the potentiometric sensor was implemented into the microfluidic cell and calibrated by using a peristaltic pump (see Figure S1 and Table S2 for the calibration graphs typically observed for the cations tested in this work).

### The Microfluidic Cell

The microfluidic cell was designed in AutoCAD 2020 (Autodesk, USA) and 3D-printed using PLA-filament using an Ultimaker 3 (Ultimaker B.V., Netherlands). The design was made for the actuator to face the sensor and provide a sample space of ca. 75 μm between them. In brief, the cell is composed of two electrode holders for the modified screen-printed electrodes. One of the electrode holders was designed with openings for attaching the fluidics (inlet and outlet). The spacer of the microfluidic cell was from VMQ elastomer (0.50 mm thick, 60 Shore A, Angst-Pfister AG, Germany), which was tailor cut using the Silhouette Cameo Cutter (USA) to achieve the microfluidic channel. The same elastomer was also tailor cut to the shape of the parts included on the 3D printed electrode holders, where on they were attached with double adhesive tape (3M 9471LE). The elastomer was attached to the electrode holder to provide a hydrophobic surface that prevented leaking from the cell. The cell was tightly closed with screws.

## Results and Discussion

**Figure 1:**
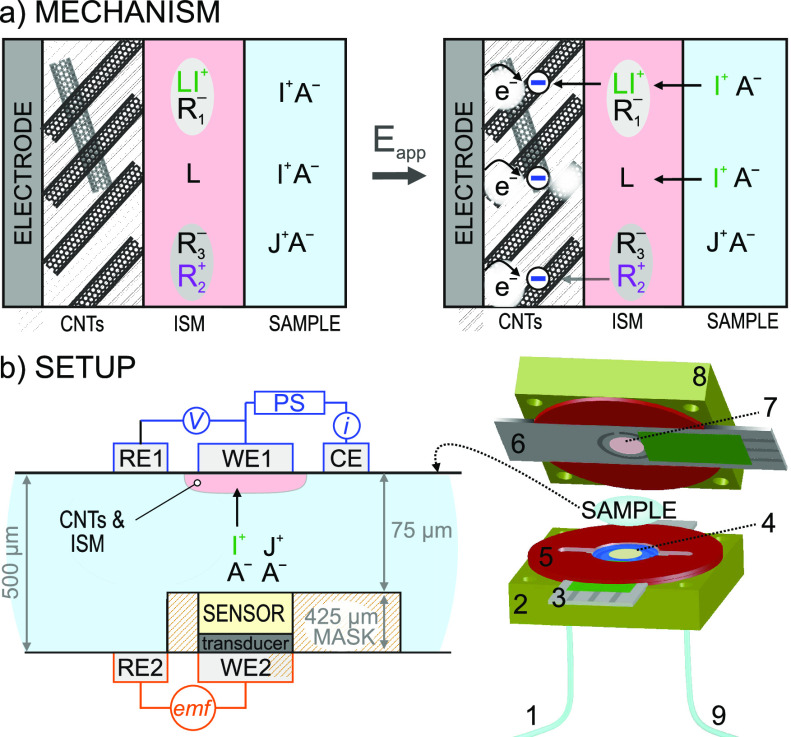
(a) Mechanism of the ion uptake caused by the application of a constant potential. A^–^, anion. I^+^ and J^+^, cations (with I^+^ being the preferred one). R_1_^–^, anion part of the cation exchange. R_2_^+^R_3_^–^, lipophilic salt. L, ionophore (selective for I^+^). ISM, ion-selective membrane. (b) The microfluidic cell. WE1, RE1, and CE indicate working, reference, and counter electrodes of the actuator. WE2 and RE2, working and reference electrodes of the potentiometric sensor. PS, power supply. emf, electromotive force. *V*, potential. *i*, current. 1, inlet; 2 and 8, electrode holders with rubber; 3 and 6, screen-printed electrodes; 4, the potentiometric sensor; 5, rubber spacer to delimit the sample; 7, CNTs and membrane in the actuator; 9, outlet.

It is herein rationalized an actuator–sensor system for the selective uptake of ions in aqueous samples, resulting in its deionization. The actuator is prepared with the CNT-ISM tandem. The ISM contains a cation ionophore (L), ion-exchanger (Na^+^R_1_^–^) and, optionally, a lipophilic salt (R_2_^+^R_3_^–^), providing a thickness of ca. 200 nm. The ISM equilibrates with the ion I^+^, for which the ionophore is selective, after ca. 20 ms of being introduced in the aqueous sample solution containing it.^18^ Accordingly, the cation in the cation exchanger (Na^+^) will be replaced by I^+^. The operating principle of the actuator is illustrated in Figure 1a. Initially, the CNTs are uncharged, and the system is in equilibrium according to the partitioning of ions between the membrane and the aqueous phase. Then, by applying a negative potential *E*_app_ with respect to the open-circuit potential (OCP), the CNTs are expected to become negatively charged. To comply with electroneutrality, an ion flux facilitated by the ionophore is generated from the solution to the ISM, while ions in the sample solution of the opposite charge are expected to diffuse/migrate toward the counter electrode. At the CNT-ISM interface, the formation of a double-layer capacitor occurred, intervening the R_2_^+^ and/or LI^+^ (ion–ionophore complex) present in the ISM.

Because of the ionophore, ideally, only I^+^ is expected to be taken up by the actuator over other cations in the solution (J^+^). This process happens in an aqueous sample confined to a thin-layer space of 75 μm (Figure 1b, left), wherein mass transport is expected to occur in ca. 3 s (see calculations in the Supporting Information). Effectively, the actuator can selectively deplete the cation concentration in the sample volume locally confined along the ISM area. The CNT-ISM actuator was set as the working electrode (WE1) in a three-electrode system, together with the reference electrode RE1 and the counter electrode CE. This configuration allows for the application of a constant potential, necessary to activate the actuator and generate the ion uptake into the membrane. The overall process is monitored by two readouts: the current decay generated at the actuator and the potential drop at the sensor. This latter can be expressed in cation concentration terms using a calibration graph.

The potentiometric sensor (WE2) contained an ISM (thicker than the membrane in the actuator) and was placed face planar to the actuator (WE1), connected to its corresponding reference electrode (RE2) for the potentiometric signal. Notably, the thickness of the sample was defined by the difference between the rubber spacer (500 μm) and the mask for the potentiometric sensor (425 μm). Thus, after assembling the cell (Figure 1b, right), all this provided a thickness of ca. 75 μm (minor uncertainties may arise from manual drop-casting of the potentiometric sensor elements).

### Investigation of the Elements Forming the Actuator

The actuator–sensor concept was first demonstrated for a 1 mM K^+^ solution, using different configurations for the actuator and a potentiometric potassium-selective electrode as the sensor to monitor the uptake process. In any case, initially, the K^+^ concentration is monitored while the solution is being pumped into the microfluidic cell. Then, the peristaltic pump was turned off, and the OCP recorded for 5 s, followed by the activation of the actuator (*E*_app_ of −0.2 V vs OCP for 120 s). Considering that K^+^ uptake is occurring, we expect to see a fast and relevant decrease in concentration from the sensor.

**Figure 2:**
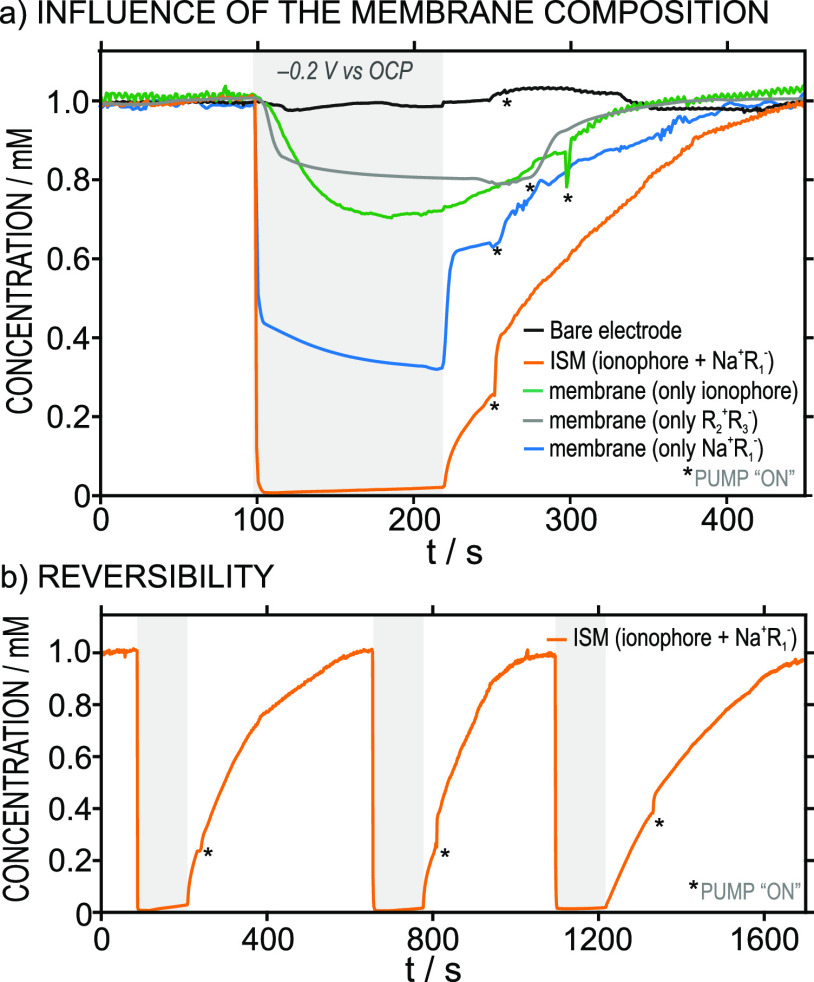
(a) Concentration profiles for K^+^ before, during, and after the activation of actuators of different compositions: bare electrode, M-I, M-XIV, M-XV, and M-XVI. (b) Three consecutive K^+^ uptakes performed with membrane M-I in the actuator. Sample: 1 mM KCl. Background electrolyte: 10 mM MgCl_2_.

When the actuator contains only CNTs (and no membrane), the described readouts are indeed observed, but any cation present in the sample will be taken up without any selectivity and following the lipophilicity order established in the Hofmeister series. For example, Figure S2 shows the concentration profiles before, during, and after the potential application for K^+^ and Na^+^. In both cases, an uptake of ca. 95% was indistinctly revealed. Importantly, the cation uptake in the solution was unequivocally provided by the charging process in the CNTs, which was assisted by those cations. Once the application of the potential ceases, such a charging process seems to be reversible, because the K^+^ concentration was found to gradually increase tending to reach the initial level. On the contrary, when the bare Dropsens electrode was used as the actuator, no change in the K^+^ concentration was detected (Figure 2a, black line).

With an actuator containing a potassium-selective membrane based on the cation exchanger (Na^+^R_1_^–^) and the K^+^ ionophore (M-I in Table S1), the concentration of K^+^ decreased in a matter of seconds from the moment that the potential was initiated until a stable concentration (0.02 mM) was reached after 10 s. This concentration was maintained as long as the potential was kept constant. Once the potential was stopped, after the 120 s of applying it, the actuator slowly released the ions back into the sample. The entire process, mainly driven by the charging/discharging of the CNTs, is reversible and could be utilized repeatedly without losing uptake efficiency (Figure 2b, 95.2 ± 2.0% of efficiency). Moreover, the regeneration process can be accomplished faster by pumping fresh solutions through the microfluidic cell, saving time in the overall analysis.

The addition of the lipophilic salt R_2_^+^R_3_^–^ to the potassium-selective membrane did not translate into a significant increase in the K^+^ uptake (efficiency of 97.9 ± 0.3%, Figure S3). Then, membranes containing only the ionophore, the lipophilic salt R_2_^+^R_3_^–^, or cation exchanger (M-XIV, M-XV, and M-XVI in Table S1, respectively) presented a considerably lower uptake compared to the case of the potassium-selective membrane (green, gray, and blue curves in Figure 2a, with 28%, 20%, and 68% of efficiency in the K^+^ uptake). The lower uptake observed for these membranes is likely due to some Mg^2+^ being taken up simultaneously to the K^+^.

In contrast to raw CNTs (Figure 2), the presence of R_2_^+^R_3_^–^ in the membrane represented an increase in the K^+^ uptake when using carboxylated CNTs (COOH–CNTs) in the actuator, as shown in our preliminary work.^17^ To better understand this difference between both types of CNTs, we performed a systematic study considering membranes containing cation exchanger together with K^+^ or Na^+^ ionophore, with or without R_2_^+^R_3_^–^ and either K^+^ or Na^+^ at a concentration of 1 mM as the ion present in the sample (together with the background electrolyte: MgCl_2_). Notably, the applied potential was different for bare CNTs and COOH–CNTs, as optimized elsewhere.^17^ In addition, two different loadings for the nanotubes were compared (0.4 mg CNTs cm^–2^ and 0.8 mg CNTs cm^–2^). The results are presented in Table 1.

**Table 1:** Results for the Uptake of K^+^ and Na^+^ in 1 mM Solutions Using Different CNT Types, CNT Loadings, and Membrane Compositions (Mainly with or without R_2_^+^R_3_^–^)^a^

Ion (ISM)	CNT Type	CNT Loading (mg/cm^2^)	*E*_app_ (V)	R_2_^+^R_3_^–^	Average Uptake ± SD (%)
K^+^ (M-I)	CNTs	0.8	–0.2	NO	95.2 ± 2.0
K^+^ (M-II)	CNTs	0.8	–0.2	YES	97.9 ± 0.3
K^+^ (M-I)	CNTs	0.4	–0.2	NO	78.1 ± 0.8^a^
K^+^ (M-II)	CNTs	0.4	–0.2	YES	75.4 ± 2.5^a^
K^+^ (M-I)	COOH–CNTs	0.4	–0.4	NO	64.0 ± 2.3^[17]^
K^+^ (M-II)	COOH–CNTs	0.4	–0.4	YES	90.6 ± 1.6^[17]^
Na^+^ (M-V)	CNTs	0.8	–0.2	NO	96.7 ± 0.9
Na^+^ (M-VI)	CNTs	0.8	–0.2	YES	95.9 ± 1.7
Na^+^ (M-V)	COOH–CNTs	0.4	–0.4	NO	91.3 ± 6.3
Na^+^ (M-IV)	COOH–CNTs	0.4	–0.4	YES	99.7 ± 0.1

a Each uptake is provided by the average and standard deviation (SD) of three consecutive measurements.

For the selective uptake of either K^+^ or Na^+^, we did not appreciate any difference when using raw CNTs (0.8 mg/cm^2^), with or without the lipophilic salt R_2_^+^R_3_^–^ in the membrane. However, for the selective uptake of Na^+^ using COOH–CNTs in the actuator (dynamic profiles shown in Figure S4), an enhanced uptake was found for the ISM with R_2_^+^R_3_^–^ (Table 1). This is similar to the results previously found for the selective K^+^ uptake using COOH–CNTs,^17^ but with a higher efficiency for the Na^+^ uptake (99.7% versus 90.6% for Na^+^ and K^+^). For COOH–CNTs, it seems clear that both the hydrophilic cation and the lipophilic R_2_^+^ counterpart participate in the CNT charge doping, whereas in the absence of R_2_^+^R_3_^–^, only hydrophilic cations participate. In addition, for the COOH–CNTs, the higher efficiency of Na^+^ uptake could be related to specific interactions with the COOH surface groups.^16^

Next, we investigated if the positive influence of R_2_^+^R_3_^–^ in the cation uptake additionally depended on the loading of CNTs in the actuator. Thus, the K^+^ uptake for a lower CNT loading (0.4 mg cm^–2^) was tested for membranes with and without R_2_^+^R_3_^–^, revealing no significant difference between them but a lower uptake than that with 0.8 mg cm^–2^. The dependence of K^+^ uptake with the CNT loading was clarified with further experiments, as shown in the next section.

Overall, the use of CNTs over COOH–CNTs displayed some advantages: there is no difference between the efficiency for K^+^ and Na^+^ using the corresponding ISM, there is no influence of the addition of R_2_^+^R_3_^–^ on the uptake efficiency and milder applied potentials are enough to obtain efficiencies of >95%. Consequently, the tandem formed by raw CNTs and an ISM without R_2_^+^R_3_^–^ was selected for subsequent experiments.

The effects of the amount of cation exchanger in the membrane and the applied potential on the ion uptake were also studied. The resulting uptakes of K^+^ (Table S3) revealed higher efficiencies at higher applied potentials, whereas the change in the concentration of the cation exchanger did not translate into significant changes. Notably, small differences in K^+^ uptake can be attributed to uncertainties inherent to the preparation of the actuator (handmade, see Experimental Section). Accordingly, a cation exchanger concentration was found to be enough to ensure at least the 98% of K^+^ uptake, while slightly increasing the magnitude of the applied potential from −0.2 V to −0.4 V improved the efficiency only by 1%.

### The Working Mechanism of the Actuator

The accumulated charge, *Q*(*t*), for the double-layer capacitor formed at the CNT-ISM interface undergoing a potential step is described by eq 1:^19^
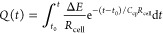
1where Δ*E* is the applied potential, *t*_0_ is the starting time of the potential step (which can be set as 0), *t* is the final time of the potential step, *R*_cell_ is the resistance of the electrochemical cell, and *C*_cp_ is the capacitance value at the CNT-ISM interface. Notably, by integration of eq 1 from *t* = 0 to *t* = ∞, eq 2 is obtained, which gives the estimated maximum charge for a potential step at the capacitor:
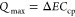
2Equation 2 is fully applicable when the electrochemical circuit does not encounter significant limitations in mass transport and the ohmic resistance is not time-dependent. This happens, for example, in a beaker experiment typically performed in benchtop electrochemistry at high electrolyte concentrations. Also, eq 2 has been applied to coulometric systems in bulk solution based on the generation of redox or double-layer capacitance at the solid contact. In such systems, the accumulated charge was correlated to the ion activity in the sample.^19−21^ However, in the case of a thin-layer solution, the resistance may change when an ion is depleted. Thus, the use of eq 2 is restricted to samples with sufficiently concentrated background electrolytes, while sometimes it permits estimations in more complex systems (e.g., thin-layer samples).

**Figure 3:**
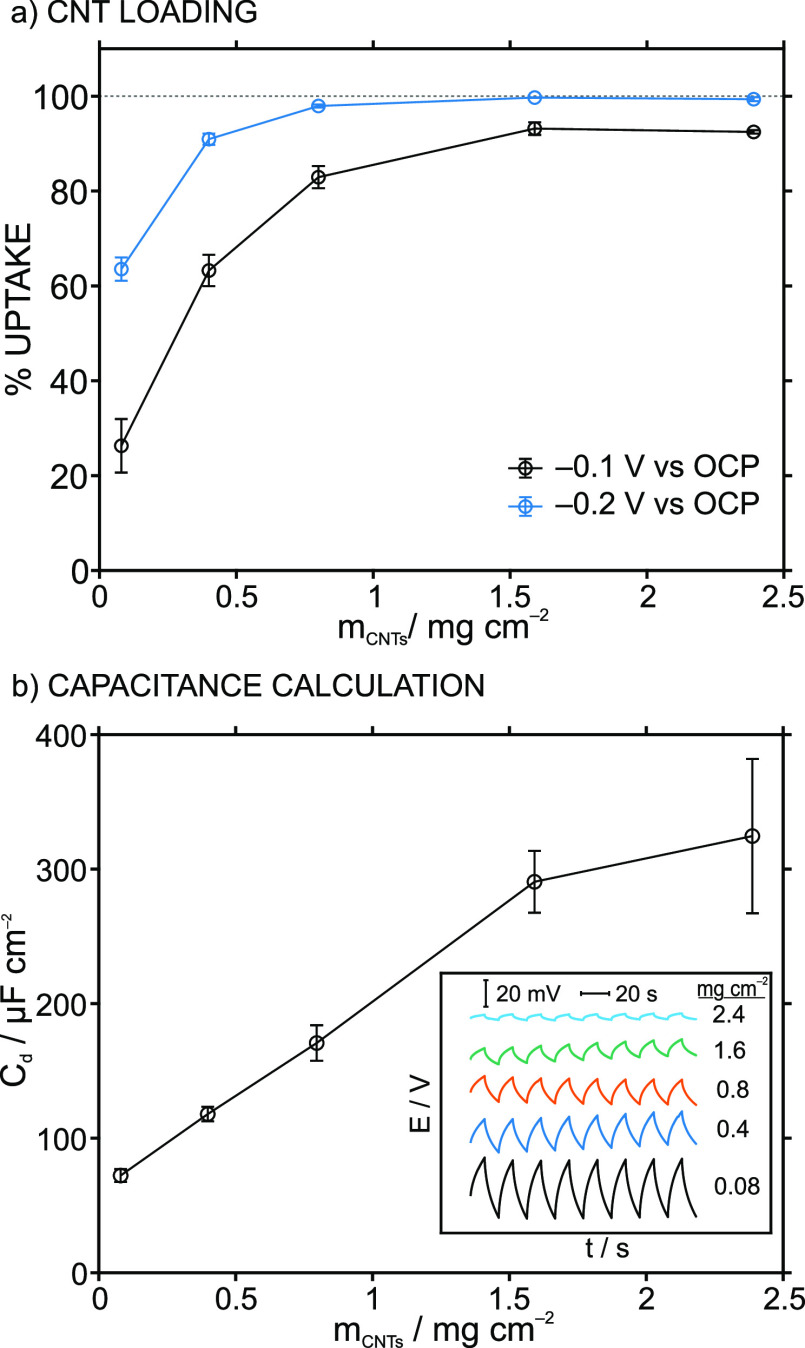
(a) The uptake percentage of K^+^ in 1 mM KCl (10 mM MgCl_2_as background electrolyte) vs increasing loading of CNTs in the actuator (*m*_CNTs_). (b) Estimated double-layer capacitance obtained for increasing loadings of CNTs in the actuator (*m*_CNTs_). The capacitance values were calculated from chronopotentiometric experiments (Inset: dynamic potential recorded at applied currents of +170 and −170 nA cm^–2^, switching every 10 s).^3^ Membrane M-I was used for the experiments..

A qualitative interpretation of either eq 1 or eq 2 concerning a double-layer capacitor (such as in the CNT-ISM actuator) implies that more charge would be accumulated at the CNTs with an increase in their capacitance or the applied potential. To confirm this behavior, the amount of CNTs drop casted onto the electrode to form the actuator was varied between 0.08 and 2.4 mg cm^–2^, and the uptake of K^+^ in 1 mM KCl was studied for two different applied potentials (see Figure 3a). Regardless of applied potential, an increase in K^+^ uptake was observed with an increase in the CNT loading, with more drastic changes appearing in the range from 0.08 to 0.8 mg cm^–2^. The K^+^ uptake at −0.1 V was always lower than that at −0.2 V for each CNT loading. Importantly, both behaviors confirmed the expectations from eq 1 and eq 2, and hence, an increase in the capacitance generated at the CNTs-ISM interface may occur with increasing CNT loading. Notably, an increase in ion uptake using CNTs (without an ISM) in deionization technology is usually explained by the increase in total capacitance.^16,22^

Bobacka and co-workers introduced a method for estimating the capacitance of an ion-to-electron transducer in contact with an ISM.^23^ This was based on chronopotentiometric measurements, where the dynamic potential *E*(*t*) acquired for a specific applied current was described by eq 3:
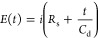
3where *R*_s_ is the bulk resistance of the ISM and *C*_d_ the low-frequency capacitance of the solid contact beneath the membrane. Notably, the derivative of eq 3 with respect to time  can be used to estimate *C*_d_. More recently, Bakker and co-workers showed that multiple chronopotentiometric measurements in succession decreased the statistical uncertainty of this method.^3^

Accordingly, we implemented an analogous procedure to estimate the capacitance associated with increasing CNT loadings covered with M-I, applying a constant current with the same magnitude but contrary sign (i.e., +170 nA cm^–2^ and −170 nA cm^–2^) every 10 s for eight cycles. These results are shown in Figure 3b: the estimated capacitance is plotted versus the CNT loading, and the corresponding chronopotentiograms are provided in the inset. As can be seen, the slope for the dynamic *E* variation (in the chronopotentiogram) was found to decrease with the CNT loading, which implies an increase in the capacitance according to eq 3.

The estimated capacitance was found to increase between 0.08 and 1.6 mg cm^–2^ CNT loading, whereafter the increase was less evident. In principle, these results imply that higher loadings of CNTs would always be preferable. However, above 1 mg cm^–2^, it was realized that the electrode surface sometimes became inhomogeneous and “flaky” during its preparation and it would easily peel off from the electrode surface. This was especially visible for the electrodes with 2.4 mg cm^–2^ CNT loading, where more than half of the actuators prepared had flakes falling off from the electrode surface. Therefore, for further studies, a loading of 0.8 mg cm^–2^ was used as a compromise between cation uptake and homogeneity of the fabricated electrodes.

Another aspect to be considered is the possibility of any contribution from impurities present in the CNT materials to the overall working mechanism of our system. For example, it has been demonstrated that the CNTs’ redox activity may be influenced by residual metal nanoparticles from the catalysts used for the CNT synthesis, which are embedded in the nanostructure.^11−13^ Thus, to discard that the K^+^ (or other cation) uptake was not caused by a redox process from impurities in the CNTs, cyclic voltammetry experiments were performed at increasing scan rates (potential window from −0.5 to 0.1 V vs the OCP) on the CNT-based actuator (Figure S5). The results revealed that only capacitive current was present.

Additionally, we carried out differential capacitance measurements in 0.1 M TBAPF_6_/acetonitrile solution (frequency = 15 Hz, signal amplitude = 10 mV, potential window from 0.8 to −0.8 V). The experimental solution was selected to mimic the lipophilic environment of a membrane phase but in a bulk domain.^3^ The results (Figure S6) showed a U-shaped curve, where the minimum represents the point of zero charge (PZC) of the CNTs (approximately at 0.03 V). The capacitance at the PZC has a value of ca. 190 μF cm^–2^, which agrees with those observed in Figure 3b. Also, the U-shaped indicates the possibility for a positive polarization of the CNTs, which implies that the CNT-ISM tandem could be also used for selective deionization of anions using anion-selective membranes (more details on PZC measurements are provided in Section 2 and eq S2 in the Supporting Information).

### Investigating the Uptake Capacity for Various Cations

**Figure 4:**
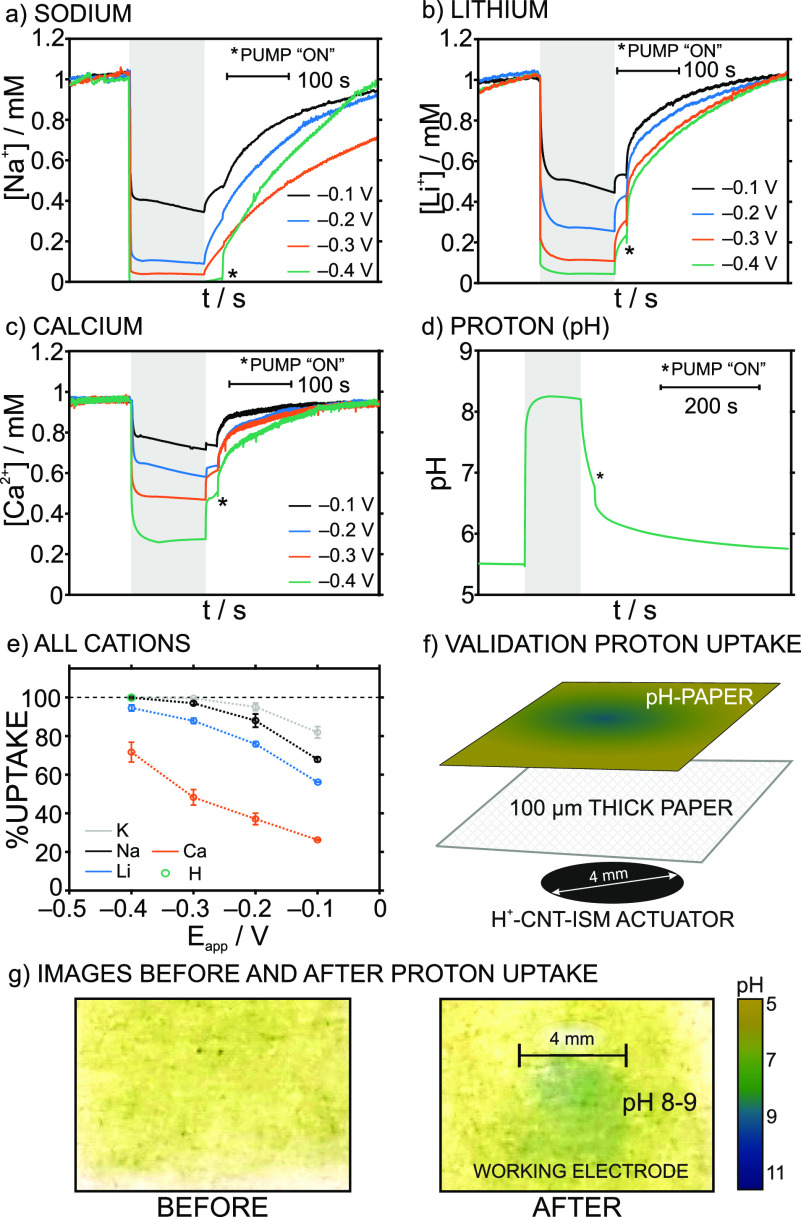
Dynamic concentrations profiles for (a) sodium, (b) lithium, (c) calcium, and (d) hydrogen ions before, during (gray area) and after the activation of the CNT-ISM tandem actuator, using membranes M-V, M-VII, M-IX, and M-XI and different potentials applied to the actuator. (e) The corresponding percentages of uptake (*n* = 3). (f) Experimental setup for validating the pH modulation measurements in 10 mM NaCl solution (initial pH of ca. 5.6). (g) Images of the pH indicator paper used in the validation of the pH modulation measurements, before and after the activation of the actuator tandem.

After recognizing the capacitive nature of the CNT-ISM tandem, we proceeded to compare the uptake of various cations (Na^+^, Li^+^, Ca^2+^, and H^+^) between −0.1 V and −0.4 V by changing the ionophore of the ISM in the actuator (M-V to M-XI in Table S1). The uptake of each cation was separately investigated in solutions with the same concentration (i.e., 1 mM) for comparison purposes (except for H^+^ which was performed in 10 mM NaCL, pH ≈ 5.6). The dynamic concentration profiles observed before, during, and after the activation of the corresponding CNT-ISM actuator are shown in Figure 4a–d. A trend similar to that displayed by K^+^ (Figure 2) was presented for all the cations. Initially, stable concentrations were read by the potentiometric sensor. Then, once the potential starts, a fast decrease (<10 s) of the cation concentration in the thin-layer sample was registered. The overall change in the cation concentration was kept as long as the polarization was activated. Finally, the concentration gradually returned to its initial levels when the potential was switched off. Such a return was sped up upon pumping new solution.

In particular for H^+^, the observed change in the sample pH was confirmed by a pH indicator paper, using the experimental setup schematized in Figure 4f. In essence, the sample was confined in a paper-based support (ca. 100 μm thick) rather than in the fluidic channel. The paper was soaked in a 10 mM NaCl solution (pH ≈ 5.6) and was then sandwiched between the CNT-ISM actuator for H^+^ and a piece of pH indicator paper. The images of the pH indicator paper before and right after stopping the activation of the actuator (−0.4 V versus the OCP, 120 s) are depicted in Figure 4g. Without any applied potential (and during 120 s), the pH indicator paper displayed no change in its color, which reflected an initial pH of ca. 5–6. After applying the potential, the pH indicator paper was found to change the color to blue-green in the area facing the working electrode, indicating an increase in the sample pH up to 8–9.

This result agrees with the uptake measured by potentiometry (Figure 4d) and confirms the capability of the CNT-ISM actuator to alkalinize the sample without the need of adding an external reagent. Notably, reagentless approaches based on solid-state materials able to modulate the sample pH are on the rise today.^24−27^ This, in combination with either optical or electrochemical sensors, has already allowed for the development of disruptive analytical concepts. Some examples where alkalinization with the CNT-ISM actuator may be of interest include precipitation of cocaine for its electrochemical detection in complex powders (dissolved in water)^27^ and speciation of nitrogen (NH_3_/NH_4_^+^) in environmental systems (e.g., waters and soils).^28^

Increasing the applied potential translated, once more, into an increase of the cation uptake (dynamic profiles for Na^+^, K^+^, and Ca^2+^ are provided in Figure 4a–c, and a comparison of the uptake percentages for all the cations are presented in Figure 4e), in agreement with the results in the previous section. However, monovalent cations presented a higher uptake than the divalent one at a similar applied potential, following the order K^+^ ≈ Na^+^ ≈ H^+^ > Li^+^ > Ca^2+^.

Importantly, the dependence of the uptake efficiency with the amount of cation-exchanger present in the membrane was further evaluated for a divalent cation, such as Ca^2+^. The utilized ISMs were M-VIII, M-IX, and M-X (Table S1), with 20, 40, and 80 mmol kg^–1^ of the cation exchanger, respectively. The dynamic profiles are depicted in Figure S7, and the uptake percentages are in Table S4. As observed, no significant differences can be made between the membrane compositions, and where the small differences observed are ascribed to uncertainties from drop-casting.^3^ Moreover, the depletion of divalent ions in the thin-layer sample was found to behave in analogy to monovalent cations, without any particular difference aside from the magnitude of the uptake percentage.

It is expected that for a given CNT-ISM configuration, a change in the ionophore nature will affect not only the preferable cation but also a change in uptake percentages according to the cation–ionophore binding constant in the ISM. In the case of the cations tested herein, Li^+^ presents the weakest binding constant with its respective ionophore (2.3 × 10^4^ kg mol^–1^), while Ca^2+^ and K^+^ possess the strongest ones (1.1 × 10^22^ kg^2^ mol^–2^ and 1.3 × 10^10^ kg mol^–1^, respectively).^29^ Nonetheless, this is not happening in our system. We have found two possible explanations, considering that diffusion in the membrane (thin-layer element) is not the limiting step.

First, the uptake may depend on the ion–ionophore stoichiometry (I^+^:L_n_), which is different for the cations tested herein: 1:1 for K^+^, Na^+^, and H^+^; 1:2 for Li^+^; and 1:3 for Ca^2+^.^29,30^ Indeed, this order coincided with that found for the uptake efficiency (Figure 4e). In such a case, increasing the amount of Li^+^ and Ca^2+^ ionophore in the corresponding membrane may equate the uptake observed for K^+^, Na^+^, and H^+^ at the same conditions. The second possibility relates to the solvation energy of the cations, with Li^+^ and Ca^2+^ being more hydrophilic than the other cations. Hence, these two will require a higher potential to be transferred from the aqueous phase to the lipophilic CNT-ISM element (more details are provided in Section 2 and eq S3 in the Supporting Information). Ionophores are known to decrease this transfer energy, but the ISM may not have enough amount of the receptor to reach such a situation. Additionally, the uptake of Ca^2+^ will involve twice the charge compared to the other cations because of its higher valency.

### Deionization Based on Membranes with Two Ionophores

Some sensing concepts may benefit from selective removal of more than one ion species at the same time. For example, potentiometric Li^+^ detection is known to suffer from strong K^+^ and Na^+^ interferences but would be advantageous in point-of-care devices for controlling Li-based treatments in Alzheimer disease. The same situation appears for NH_4_^+^ detection in biofluids, and in environmental waters.^31^ Also, it is known that the high salt content in seawater masks the detection of other ions (specially at trace levels); therefore, seawater analysis would profit from a predesalination step (e.g., simultaneous removal of Na^+^ and Cl^–^, or other ion pairs).^32^

**Figure 5:**
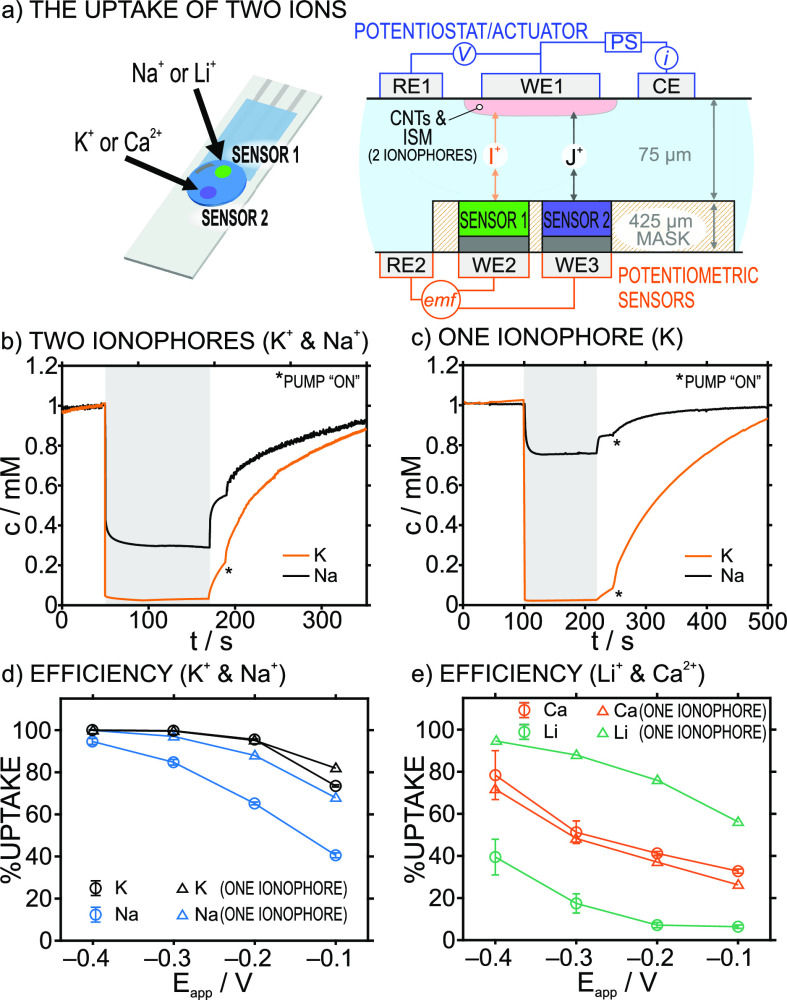
(a) Diagrams of sensors and setup for the experiments involving the uptake of two ions with CNT-ISM actuators based on two ionophores. Dynamic concentration profiles before, during (gray area) and after the uptake for (b) K^+^ and Na^+^ using M-XII (two ionophores) in the CNT-ISM actuator and (c) the uptake of K^+^ and Na^+^ using M-I (potassium ionophore) in the CNT-ISM actuator (−0.2 V versus OCP), in sample: 1 mM NaCl/1 mM KCl. The uptake percentage of two ionophores ISMs are presented (d) for K^+^ and Na^+^ (M-XII) and (e) for Li^+^ and Ca^2+^ (in 1 mM LiCl/1 mM CaCl_2_), at different applied potentials (circles) compared to the one ionophore uptakes (triangles). Error bars represent the standard deviation (*n* = 3). Background electrolyte: 10 mM MgCl_2_.

A CNT-ISM actuator containing two ionophores was therefore developed and tested. In particular, the ISM was based on equimolar concentrations of K and Na ionophores and the cation exchanger (membrane M-XII, Table S1). To ensure the simultaneous monitoring of the two cations, the sensor in the microfluidic cell was modified: a screen-printed electrode with two working electrodes independently modified with potentiometric ISMs to detect K^+^ and Na^+^ (Figure 5a, left). Thus, the thin-layer sample is sandwiched between the CNT-ISM actuator (RE1, WE1, and CE in Figure 5a, right) containing two ionophores, and the two potentiometric sensors for K^+^ and Na^+^ (WE2 and WE3) facing the actuator, sharing a common reference electrode (RE2) (Figure 5a, right).

Using the described system, the dynamic concentration profiles resulting from the uptake of both K^+^ and Na^+^ (at −0.2 V versus the OCP) in a solution containing 1 mM KCl + 1 mM NaCl are displayed in Figure 5b. Interestingly, the K^+^ uptake presented the same efficiency (95%) as that obtained with only one ionophore in the membrane (Figure 5c), while simultaneously removing ca. 60% of the Na^+^ in the sample. If the Na ionophore had not been included in the CNT-ISM actuator, only ca. 20% of Na^+^ would have been taken up (Figure 5c). When using the two ionophores in the actuator, the lower uptake of Na^+^ compared to K^+^ was maintained regardless of the applied potential (Figure 5d, circles). However, at −0.4 V, the removal of both cations from the sample was higher than 90%, approaching more to what was obtained using individual ionophores for K^+^ or Na^+^ in the actuator (Figure 5d, triangles).

The K ionophore is known to exhibit a stronger bond with K^+^ compared with that for the Na ionophore (1.3 × 10^10^ kg mol^–1^ and 4.9 × 10^7^ kg mol^–1^ for the binding constants).^29^ This suggests that, in our experiments, the selective deionization using two ionophores is controlled not only by the applied potential but also by the strengths to bind the ions provided by the ionophores. To further test this hypothesis, a membrane containing two ionophores presenting a larger difference between their binding constants was tested, based on Li and Ca ionophores, with 2.3 × 10^4^ kg mol^–1^ and 1.1 × 10^22^ kg^2^ mol^–2^ for the binding constants (M-XIII, Table S1).^29^ The resulting uptakes at different applied potentials are presented in Figure 5e, with some selected dynamic concentration profiles being displayed in Figure S8. For comparison purposes, the results provided by the actuators based on either the Ca or Li ionophores are also presented in Figure 5e (triangles). As observed, the uptake of Ca^2+^ does not change whether one or two ionophores are used in the CNT-ISM tandem. However, the uptake of Li^+^ was found to be below 10% for potentials lower than −0.3 V (versus the OCP). These results confirm that the binding strengths of the ion–ionophore pairs govern the preference of the CNT-ISM tandem regarding the ion that will be removed from the sample when having equimolar ionophore concentrations in the membrane.

Despite the stronger ionophore controlling the main ion for deionization, the uptake degree for two ions can additionally be adjusted by increasing the magnitude of the applied potential. Moreover, the molar ratio of the two ionophores in the membrane could also be investigated for a more efficient uptake of the two respective ions, which will be necessary to explore when meeting applications of the CNT-ISM actuators in real samples. Overall, both one or two ionophore-based ISMs for selective deionization of thin-layer samples are foreseen to be implemented in multiple analytical devices, including the detection of Li^+^ in biofluids (with Na^+^ and K^+^ as the main interferents)^33^ and Mg^2+^ in biofluids or environmental water (with H^+^ and Ca^2+^ as the main interferents),^34^ among other cases.

## Conclusions

It is herein demonstrated that the mechanism of CNT-ISM tandems providing selective deionization of thin-layer samples at a constant applied potential is of a capacitive nature. The degree of deionization was found to be related to the capacitance at the (solid-contact) CNT-ISM interface, controlled by the CNTs loading in the actuator. On the other hand, the efficiency of the total uptake was found to be independent of the concentration of cation-exchanger in the membrane. However, when CNTs modified with COOH groups were used in the actuator, higher uptake efficiencies were found in the presence of an additional lipophilic salt in the membrane. This is probably because of specific interactions between the cations and the COOH groups. The relevance of this concept from an analytical perspective was enlightened by investigating the selective uptake of five different ions (K^+^, Na^+^, H^+^, Li^+^, and Ca^2+^). Moreover, by introducing two ionophores in the ISM forming the actuator, two ions can be simultaneously removed from the thin-layer sample. It was found that this process depends on the strength of the ion–ionophore interactions and the magnitude of the applied potential. This work demonstrates the great versatility for further opportunities of the principle of selective deionization with a CNT-ISM tandem, opening a path for its integration into analytical devices requiring in situ removal of interferent ions to accurately detect primary analytes.

## Supporting Information Available

Experimental section, additional calculations, sensor calibrations, tables of ISM-compositions and ion-uptakes, and voltammetry data (PDF)
