# Supplementary material for: Selective Deionization of Thin-Layer Samples Using Tandem Carbon Nanotubes–Polymeric Membranes
Source: Anal Chem. 2023 Oct 10;95(42):15681–9. doi: 10.1021/acs.analchem.3c02965 (PMC10603610; doi:10.1021/acs.analchem.3c02965)
Supplement: Supplementary file 1 — ac3c02965_si_001.pdf [file ac3c02965_si_001.pdf]

Supporting Information for:

Selective Deionization of Thin-Layer Samples using the Tandem Carbon Nanotubes – Polymeric Membranes

Alexander Wiorek[a], Maria Cuartero[a,b*] and Gaston A. Crespo[a,b*]

aDepartment of Chemistry, School of Engineering Science in Chemistry, Biochemistry and Health, KTH Royal Institute of Technology, Teknikringen 30, SE-11428, Stockholm, Sweden

[a]UCAM-SENS, Universidad Católica San Antonio de Murcia, UCAM HiTech, Avda. Andres Hernandez Ros 1, 30107, Murcia, Spain

Corresponding authors (*): gacp@kth.se ; mariacb@kth.se

Table of Contents

1. **Experimental Section** 2

Reagents and materials.....2

Instrumentation to control the actuator-sensor system 2

2. **Calculations and additional comments on experimental results** 3

Estimated time for ion uptake......3

Differential Capacitance measurement and The Point of Zero Charge. 3

The energy of ion-transfer. 3

3. **Tables** 4

Table S1 4

Table S2 4

Table S3 5

Table S4 5

4. **Figures** 6

Figure S1 6

Figure S2 7

Figure S3 7

Figure S4 8

Figure S5 8

Figure S6 9

Figure S7 9

Figure S8 10

5. **References**.....11

1. Experimental Section

Reagents and materials. High molecular weight poly(vinyl chloride) (PVC), potassium ionophore I (valinomycin), sodium ionophore X (4-tert-butylcalix[4]arene-tetraacetic acid tetraethyl ester), hydrogen ionophore I (tridodecylamine), lithium ionophore VI (6,6-Dibenzyl-14-crown-4), calcium ionophore IV (ETH 5234), chromoionophore I (ETH 5294), sodium tetrakis[3,5-bis-(trifluoromethyl)phenyl]borate ($Na^+R_1^-$), tetradodecylammonium tetrakis(4-chlorophenyl)borate (ETH500, $R_2^+R_3^-$), bis(2-ethylhexyl)sebacate (DOS), tetrahydrofuran (> 99.9%, THF), tetrahydrofuran (THF), potassium chloride (99.5%, KCl), sodium chloride (99.5%), calcium chloride (≥99.0%), potassium phosphate dibasic (99%), potassium phosphate monobasic (99%), sodium carbonate (≥99.5%), acetic acid (≥99%), sodium acetate (99%), sulfuric acid (99% w/w), and Trizma Base® (99.9%), were purchased from Sigma Aldrich. Hydrochloric acid (reagent grade, 1 M), sodium bicarbonate (99.9%), and sodium hydroxide was purchased from VWR chemicals. Magnesium chloride (99%) was purchased from Alfa Aesar. The CNTs used, unless otherwise specified, were Multi-walled Carbon Nanotubes (outer diameter 30-50 nm, length 50 µm, purity>95 wt%, ash>1.5 wt%, specific surface area>60 m2/g) purchased from HeJi. All the solutions were prepared with ultrapure water, resistance of 18.2 MΩ cm (Milli-Q water systems, Merck Millipore).

Instrumentation to control the actuator-sensor system. Electrochemical experiments were performed using a PGSTAT302 Autolab potentiostat (Metrohm Nordic AB) operated using the Nova 2.1.5 software on a PC. The EMF for the potentiometric sensors was recorded using a high input impedance (1015Ω) EMF16 multichannel data acquisition device, Lawson labs EMF16 Interface (Lawson Laboratories, Inc.). The pH was measured using a 914 pH/Conductometer from Metrohm (6.0228.000). For screen printed electrodes, either a Dropsens DRP150 was used for mono-ionophore based ISMs (actuators and potentiometric sensors), or a Dropsens DRP1110 electrode for two-ionophore experiments (potentiometric sensors).

2. Calculations and additional comments on experimental results

Estimated time for ion uptake. The time of diffusion t over a certain distance x in one dimension can be estimated by **Equation S1**.

$$t = \frac{\langle x \rangle^2}{2D} \tag{S1}$$

Where D is the diffusion coefficient of the phase. The diffusion coefficient is be estimated to be ca. 10^{-5} cm^2/s for an aqueous phase,[1] and ca. 10^{-8} cm^2/s for a membrane phase.[2] Using **Equation S1** for a thin-layer sample (aqueous) of 75 µm, the time of diffusion for an ion through it becomes 2.8 s. For the membrane phase used in this this work (ca 200 nm of thickness) the time becomes 20 µs. Thus, diffusion through the membrane phase is not expected to limit the rate of the ion uptake process.

Differential Capacitance measurement and The Point of Zero Charge. The point of zero charge (PZC) was studied between -0.8 and 0.8 V in acetonitrile with $TBAPF_6$ as background electrolyte with the signal amplitude of 10 mV (**Figure S6**). The capacitance C_d was then calculated from the angular frequency ($2\pi f$, where f is the frequency; 15 Hz) and the imaginary component of the impedance, given in **Equation S2**.

$$C_d = -\frac{1}{2\pi f Z''} \tag{S2}$$

This revealed the U-shape curve observed in **Figure S6** which is characteristic for a purely capacitive mechanism.[3]

The PZC indicates the potential where the surface charge is zero, where at higher- and lower potentials the surface charge is positive and negative, respectively.[3] This gives an indication that the surface charge is not limited for negative polarization for storage of cations in the double-layer, but indicates the possibility to charge the CNTs positively for anion uptake. This has indeed been utilized without membranes in desalination technologies aimed for drinking water applications,[4] and could likely be explored in the future for selective uptake of anions in analytical applications using ISMs based on anion-selective ionophores (such as for Cl^- uptake).

The energy of ion-transfer. The energy for transferring an arbitrary ion from one phase [1] to another [2] is given by **Equation S3**.[5]

$$\Delta\mu^i = -\frac{z^2 e^2}{8\pi\epsilon_0 a}\left[\frac{1}{\epsilon_1} - \frac{1}{\epsilon_2}\right] \tag{S3}$$

where $\Delta\mu^i$ is the difference in solvation energy between the two phases, z is the valence charge, e the elementary charge, ϵ_0 the permittivity of free space, a the ion radius and ϵ_1 and ϵ_2 the dielectric constants of the respective phases.

Examining **Equation S3** in the case of ion-transfer from water to the ISM phase, the energy of transfer is positive ($\epsilon_1 > \epsilon_2$, as water has a higher dielectric constant than DOS and PVC)[5]. Additionally, the energy of transfer increases by the square of the charge and increase by $\frac{1}{a}$ for decreasing ion-radius. Thus, the energy of transferring Ca^{2+} and Li^+ into the CNTs are expected to be larger than that of larger, monovalent ions (such as K^+ and Na^+).

3. Tables

Table S1. Membrane compositions. All the membranes were prepared with 1:2 mass ratio of PVC:DOS.

ISM-Compositions / mmol kg^{-1}				
ISM	Selectivity	Ionophore	Ion Exchanger	ETH500
M-I	K	80	40	-
M-II	K	80	40	40
M-III	K	80	20	-
M-IV	K	80	80	-
M-V	Na	80	40	-
M-VI	Na	80	40	40
M-VII	Li	80	40	-
M-VIII	Ca	80	20	-
M-IX	Ca	80	40	-
M-X	Ca	80	80	-
M-XI	H	20	10	-
M-XII	K & Na	80 (L-K) / 80 (L-Na)	80	-
M-XIII	Li & Ca	80 (L-Li) / 80 (L-Ca)	80	-
M-XIV	K	80	-	-
M-XV	-	-	-	40
M-XVI	-	-	40	-

Table S2. Calibration parameters obtained from the different potentiometric sensors, where the corresponding sensitivities and intercepts are given as the average with standard deviation for seven different sensors (n=7).

Sensor	ISM	Sensitivity / mV dec^{-1}	Intercept / mV	Linear Range / M
K	II	60.1±2.0	588.6±16.7	10^{-6}-10^{-2}
Na	V	59.1±1.4	361.1±.29.3	10^{-6}-10^{-2}
Li	III	60.9±1.1	302.1±17.0	10^{-5}-10^{-2}
Ca	IV	30.3±1.8	303.2±61.6	10^{-6}-10^{-2}
H	XII	−55.5±0.5[a]	522.5±44.5	(pH) 2.2-10.6

[a]mV/pH,

Table S3. Results for the uptake of K^+ varying the concentration of the cation exchanger in the membrane and using different applied potentials. Experiments were performed in 1 mM KCl solution with 10 mM $MgCl_2$ as the background electrolyte. Each uptake is provided by the average and standard deviation of three consecutive measurements.

ISM	Cation Exchanger (mmol kg^{-1})	Average Uptake (%)		
		–0.1 V	–0.2 V	–0.4 V
M-III	20	77±1	96.0±0.7	99.92±0.03
M-I	40	82±3	98.3±0.7	99.98±0.02
M-II	80	78±2	95.9±0.5	99.85±0.01

Table S4. The uptake of Ca^{2+} for different concentrations of ion-exchanger (NaTFPB) in the membrane (n=3), with an applied potential of –0.4 V versus the OCP.

ISM	Ion Exchanger / mmol kg^{-1}	Ca^{2+} Uptake / %
VIII	20	83±6
IX	40	72±5
X	80	84±7

4. Figures

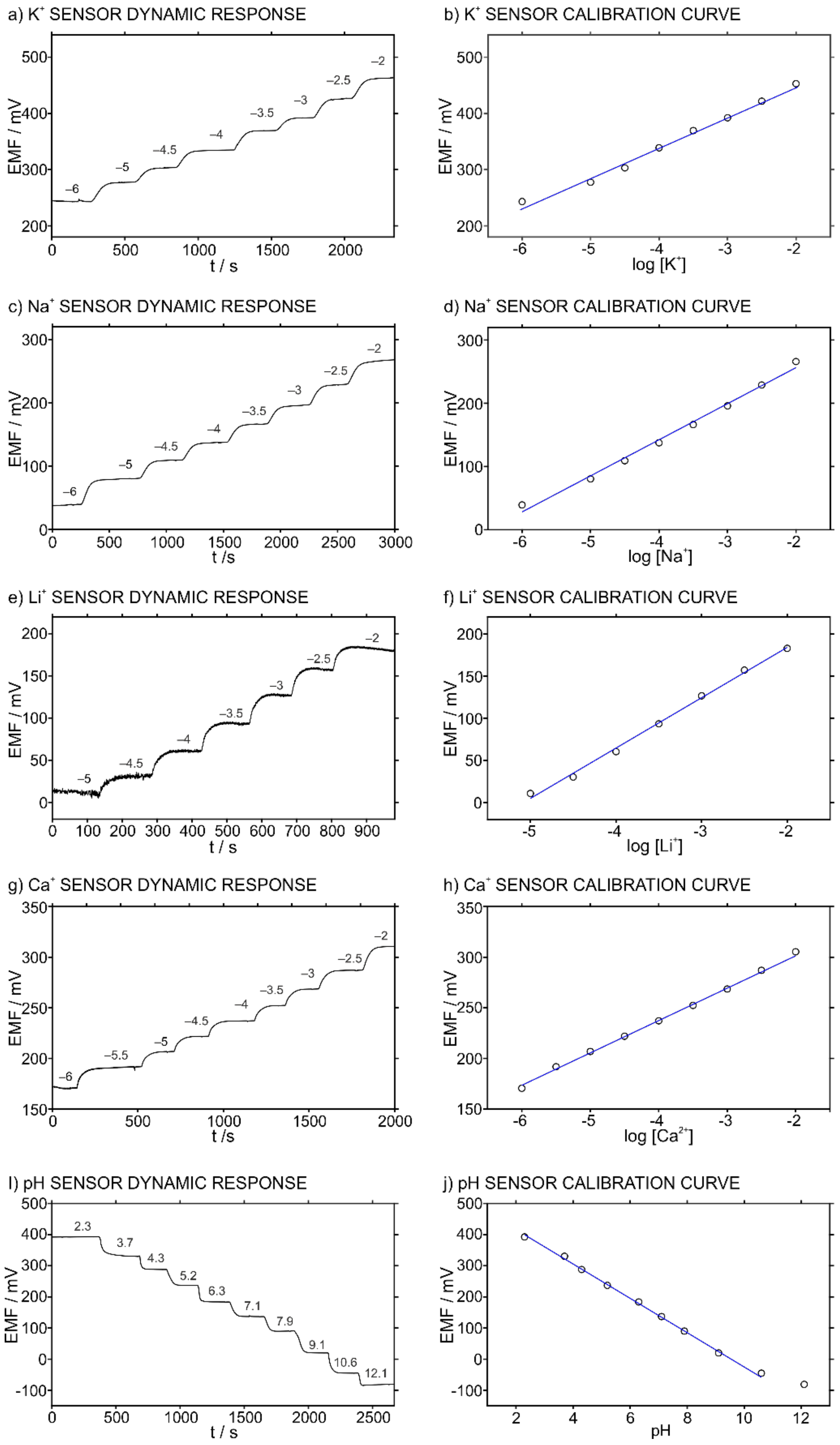

Figure S1. Calibrations of the different potentiometric sensors used in this work where a), c) e), g) and i) presents the dynamic responses from calibrations of a K, Na, Li, Ca and pH sensors, respectively. Their corresponding calibration curves are plotted in b), d), f), h) and j). Calibrations were performed in the microfluidic cell, with a pump-rate of 100 μL/min.

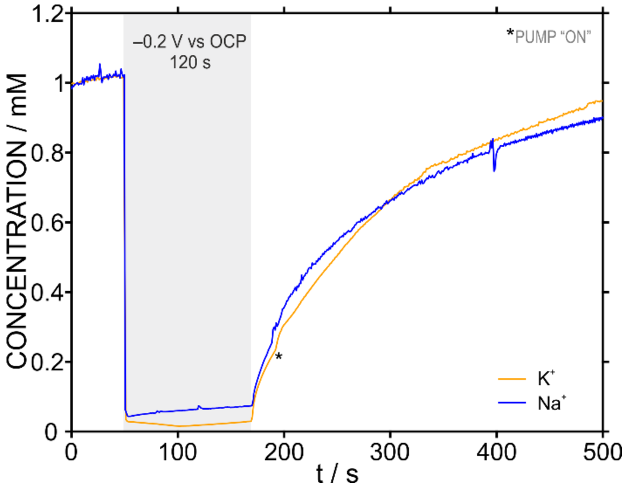

Figure S2. Uptake of K^+ and Na^+ without an ISM on the actuator (i.e., only CNTs) in 1 mM KCl or 1 mM NaCl solution with 10 mM $MgCl_2$ as background electrolyte.

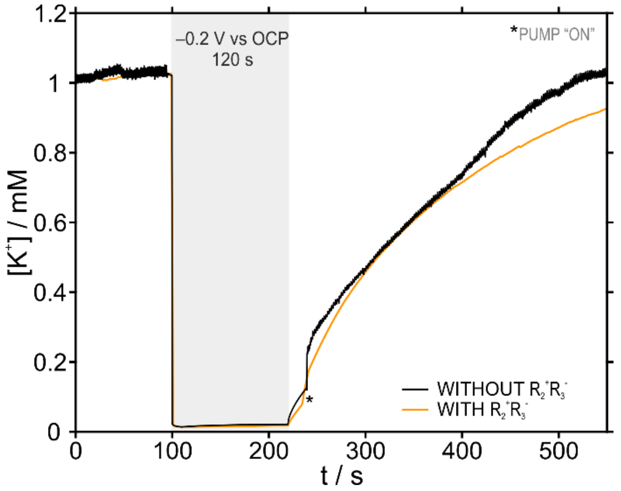

Figure S3. Comparison between uptakes of K^+ using ISMs without $R_2^+R_3^-$ (M-I) and with $R_2^+R_3^-$ (M-II). Sample: 1 mM KCl. Background electrolyte: 10 mM $MgCl_2$.

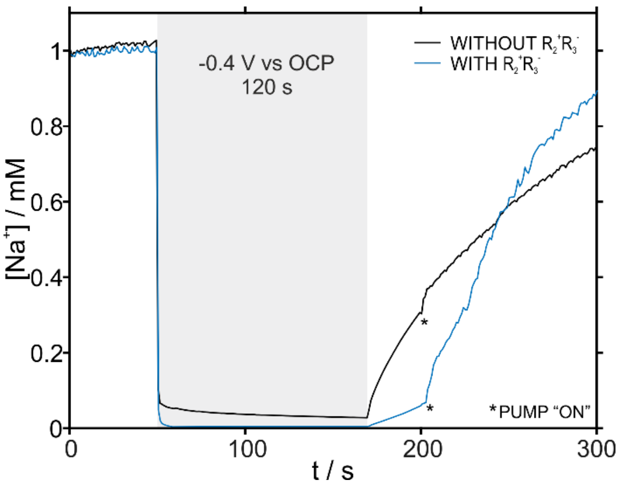

Figure S4. Examples showing the uptake of Na^+ using COOH-CNTs and membranes with or without $R_2^+R_3^-$ (ISM V and VI, Table S1) at –0.4 V versus the OCP. The experiments were performed in 1 mM NaCl solution with 10 mM $MgCl_2$ as the background electrolyte.

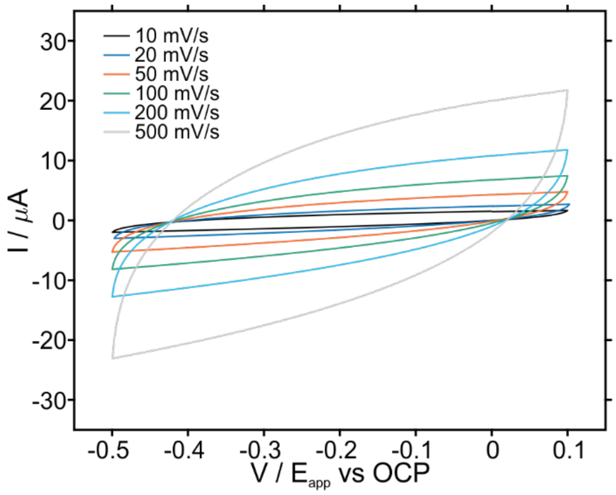

Figure S5. Cyclic voltammograms of the CNTs (0.80 mg cm^{-2}) in a beaker with 1 mM KCl solution (10 mM $MgCl_2$ as the background electrolyte). The initial OCP was measured to 0.31 V (i.e., at 0 V in the voltammogram).

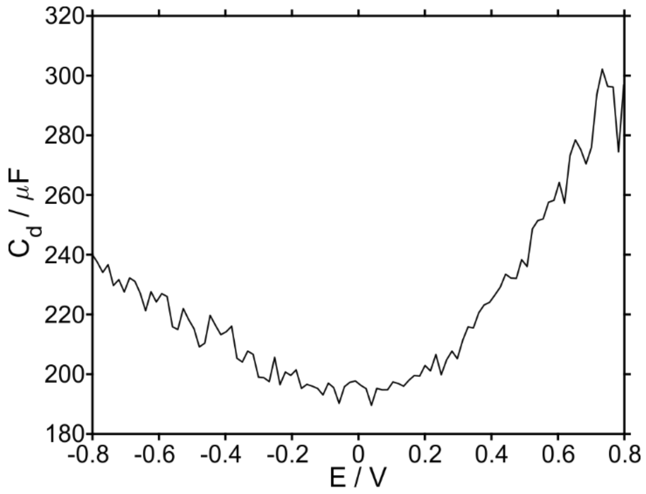

Figure S6. Differential capacitance measurements of the CNTs in 0.1 M $TBAPF_6$ / acetonitrile solution. The measurements were conducted at 15 Hz, from 0.8 to -0.8 V vs the Ag/AgCl pseudo reference electrode. At this frequency, the contribution of any faradaic processes is negligible within the potential window (i.e., only capacitive contributions are expected in the differential capacitance).

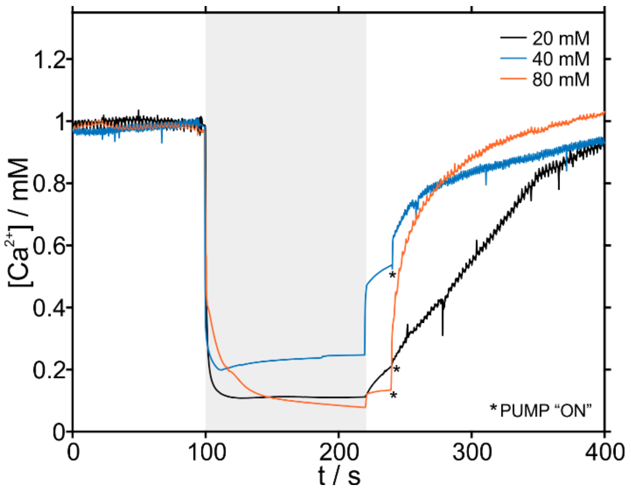

Figure S7. Examples of the dynamic uptake of Ca^{2+} for different concentrations of the cation exchanger in the membrane. The gray area represents the time over which the potential was applied to the actuator (−0.4 V versus the OCP, 120 s). Experiments were performed in 1 mM $CaCl_2$ solution with 10 mM $MgCl_2$ as the background electrolyte.

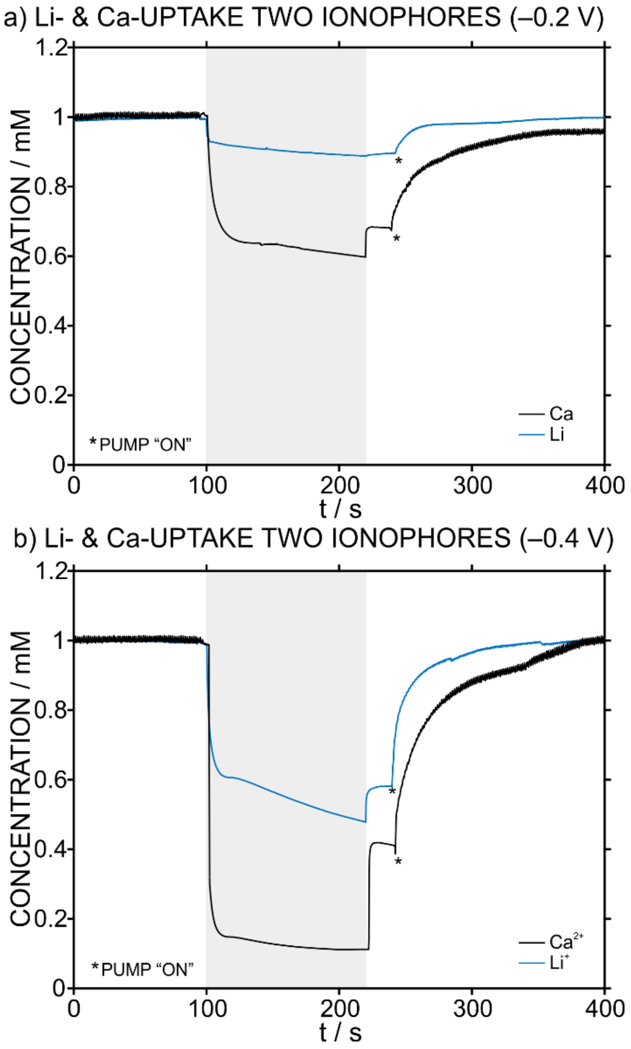

Figure S8. Uptake of Li^+ and Ca^{2+} using an ISM with two ionophores (M XIII) at an applied potential of a) –0.2 V versus the OCP and b) –0.4 V versus the OCP. The sample was 1 mM LiCl/1 mM $CaCl_2$ with 10 mM $MgCl_2$ as the background electrolyte.

5. References

1. Gosting, L. J., A Study of the Diffusion of Potassium Chloride in Water at 25° with the Gouy Interference Method. *Journal of American Chemical Society* **1950,** *72* (10), 4418–4422.
2. Lindfors, T.; Sundfors, F.; Höfler, L.; Gyurcsányi, R. E., FTIR-ATR Study of Water Uptake and Diffusion Through Ion-Selective Membranes Based on Plasticized Poly(vinyl chloride). *Electroanalysis* **2009,** *21* (17-18), 1914-1922.
3. Bard, A. J.; Faulkner, L. R., *Electrochemical Methods Fundamentals and Applications*. 2nd ed.; John Wiley & Sons, Inc: 2001.
4. Wang, R.; Chen, D.; Wang, Q.; Ying, Y.; Gao, W.; Xie, L., Recent Advances in Applications of Carbon Nanotubes for Desalination: A Review. *Nanomaterials* **2020,** *10* (6), 1203.
5. Israelachvili, J. N., *Intermolecular and surface forces*. 3rd ed. ed.; Academic Press: Burlington, Massachusetts, 2011.
